# Establishing a novel mouse model of tacrolimus-induced post-transplant hepatocellular carcinoma pulmonary recurrence for transplant oncology

**DOI:** 10.3389/fcell.2026.1796566

**Published:** 2026-04-01

**Authors:** Jinliang Duan, Tao Chen, Shaofeng Chen, Zhenglu Wang, Lei Cao, Hong Zheng, Zhongyang Shen

**Affiliations:** 1 Nankai University School of Medicine, Tianjin, China; 2 Tianjin Organ Transplantation Research Center, Tianjin First Central Hospital, Tianjin, China; 3 Biological Sample Resource Sharing Center, Tianjin First Central Hospital, Tianjin, China; 4 Institute of Transplantation Medicine, Nankai University, Tianjin, China; 5 Tianjin Key Laboratory of Organ Transplantation, Tianjin First Central Hospital, Tianjin, China; 6 Key Laboratory of Transplant Medicine, Chinese Academy of Medical Science, Tianjin, China

**Keywords:** allotransplantation, cancer recurrence, hepatocellular carcinoma, immunosuppression, mouse model

## Abstract

**Background:**

Long-term immunosuppression following transplantation places recipients at a high risk of malignancy. Hepatocellular carcinoma (HCC) recurrence after transplantation poses significant challenges to long-term survival of recipients. Several studies in transplant oncology have established cancer-transplant models to support the development of therapies for reducing the risk of post-transplant cancer recurrence. However, existing models fail to recapitulate the complex immune status of recipients and the authentic tumor microenvironment after transplantation.

**Methods:**

C57BL/6 recipient mice were injected intravenously with luciferase-expressing Hepa1-6 cells 7 days before receiving Balb/c cardiac allografts, followed by post-transplant immunosuppression with tacrolimus. On day 7 post-transplantation, the immune state of recipients was assessed by measuring serum inflammatory cytokines, and allograft rejection was evaluated by hematoxylin-eosin staining. Cancer progression was evaluated using *in vivo* imaging and measurements of serum alpha-fetoprotein levels, with subsequent histological confirmation.

**Results:**

Allogeneic cardiac transplantation resulted in a significant increase in serum levels of pro-inflammatory cytokines accompanied by a marked reduction in cancer burden. Marked lymphocytic infiltration, hemorrhage, and structural disintegration were observed in the grafts of untreated animals. In contrast, tacrolimus treatment effectively attenuated both the inflammatory cytokine response and acute allograft rejection but, conversely, resulted in a significant increase in tumor burden. Histological analysis confirmed that malignancies were exclusively localized to the lungs, mirroring the most common site of clinical HCC recurrence post-transplantation.

**Conclusion:**

This model effectively simulates the elevated risk of cancer recurrence under post-transplant immunosuppression and faithfully recapitulates lung metastasis of HCC mediated by circulating cancer cells—the most common site of clinical recurrence following liver transplantation for HCC. It thus provides a reliable tool for basic research aimed at reducing risk of post-transplant cancer recurrence.

## 1 Introduction

As the fourth leading cause of global cancer-related death, hepatocellular carcinoma (HCC) is unlike other cancers in that its overall burden has been on the rise worldwide (Zhang et al., 2024). Liver transplantation serves as a recognized curative treatment for HCC, particularly in patients with small, unresectable HCC accompanied by cirrhosis (Mazzaferro et al., 1996). Although first-line immunosuppressants, such as tacrolimus (FK506), are highly effective in attenuating rejection, they also suppress the recipient’s immune cells non-specifically. There is an inherent conflict between the long-term immunosuppressive state required to prevent allograft rejection and the process of tumor immune surveillance. The impairment of tumor immune surveillance significantly increases the risk of HCC recurrence, which represents a major obstacle to long-term survival in recipients (Rodríguez-Perálv et al., 2022). The 5-year recurrence rate for HCC after liver transplantation ranges from 20% to 57.8% (Duvoux et al., 2012), and remains as high as 10% even under the strictest Milan Criteria (Mazzaferro et al., 2009). Once HCC recurs, the median survival time of recipients is only ranges from 10.6 to 12.2 months (Bodzin et al., 2017). Therefore, controlling the risk of post-transplant cancer recurrence represents a critical challenge and an urgent need in clinical liver transplantation for HCC.

Transplant oncology is an emerging field that integrates oncology and immunology to address the inherent conflict between transplantation and cancer (Krendl et al., 2024). In this discipline, preventing post-transplant malignancies and improving long-term survival for high-risk patients play a critical role (Kong et al., 2024a). To elucidate the mechanisms and develop preventive strategies for post-transplant HCC recurrence, several cancer-transplant animal models have been established. For instance, liver transplantation in a HCC animal model can mimic the natural recurrence of HCC after clinical transplantation, but the model establishment is inherently time-consuming and variable even under immunosuppression (Matsuzaki et al., 1992; Yan et al., 2013; Schotman et al., 1998; Li et al., 2004; Ceriello et al., 1994; Yano et al., 1998). Qi et al. performed syngeneic liver transplantation in Buffalo rats and injected McA-RH7777 cells into the recipient’s portal vein following blood flow restoration (Qi et al., 2016). The syngeneic transplantation model does not capture the critical role of immunosuppressed state in HCC recurrence following liver transplantation. Yang et al. subcutaneously injected Hepa1-6 cells into recipient mice prior to transplanting Balb/c skin grafts into C57BL/6 mice (Yang et al., 2023). However, skin transplantation lacks the vascular-associated rejection and functional impairment characteristic of vascularized organ transplantation. The abdominal heterotopic mouse heart transplantation model represents a classic experimental system in transplant immunology. It is not only highly stable and reproducible but also facilitates straightforward assessment of graft survival. Moreover, this model closely recapitulates the immune responses observed following clinical vascularized organ transplantation. The subcutaneous tumor-bearing mouse heart transplantation model, reported by Guba, was once considered an important tool in transplant oncology research (Koehl et al., 2004). However, subcutaneous tumors lack an authentic tumor microenvironment, and drugs effective in such models demonstrate low success rates when translated to clinical applications (Jiao et al., 2019). Collectively, existing cancer-transplant models are compromised by deficiencies in the following key aspects: stability, a vascularized allograft, the immunosuppressed state, and an authentic tumor microenvironment.

In clinical practice, the lungs represent the most common site of cancer recurrence following liver transplantation for HCC, occurring in 50%–60% of cases, followed by the liver graft, abdominal cavity, bones, and other locations (Bodzin et al., 2017; Alshahrani et al., 2018; Na et al., 2016; de'Angelis et al., 2015). Experimental lung metastasis model of HCC is commonly established by injecting HCC cells via the tail vein, leading them to travel through the systemic circulation and first lodge in the lungs (Luo et al., 2024). The signal intensity obtained from *in vivo* imaging exhibits a strong correlation with the number of luciferase-expressing tumor cells inoculated into mice, making this technique particularly suitable for monitoring these engineered cells *in vivo* (Tseng and Kung, 2015). Furthermore, HCC-specific tumor biomarkers can serve as indicators of tumor burden (Zhou et al., 2023).

In this study, we established an FK506-mediated mouse model of post-transplant HCC pulmonary recurrence. This model faithfully recapitulates the clinical scenario of HCC recurrence after liver transplantation, thereby providing a reliable platform for developing strategies to prevent this serious complication.

## 2 Methods

### 2.1 Animals and groups

C57BL/6J (H2b) and BALB/c (H2d) mice, which served as recipients and donors respectively, were obtained from Beijing Vital River Laboratory Animal Technology Co. Ltd. (Beijing, China). All mice were 8–10 weeks old and weighed 22–25 g at the commencement of the studies. All experimental procedures were performed in accordance with the ARRIVE criteria (Kilkenny et al., 2010) and approved by the Animal Ethics Committee of Genink Biotechnology Co., Ltd. (Tianjin, China). The registration number of the ethical approval is GENINK-20250073.

A total of five experimental groups were included in this study, with all mice randomly assigned to each group. (Zhang et al., 2024). HCC group: Mice were intravenously injected via the tail vein with 2 × 10^6^ luciferase-expressing Hepa1-6 (Hepa1-6 luc) cells. (Mazzaferro et al., 1996). ALLO group: Mice received an intravenous injection of an equal volume of PBS via the tail vein, followed 7 days later by heterotopic heart transplantation from a Balb/c donor. (Rodríguez-Perálv et al., 2022). HCC + ALLO group: Mice were injected intravenously with 2 × 10^6^ Hepa1-6-luc cells via the tail vein and, after 7 days, underwent heterotopic heart transplantation from a Balb/c donor. (Duvoux et al., 2012). HCC + ALLO + FK506 group: Based on the HCC + ALLO group procedures, mice additionally received a daily oral gavage of FK506 (3 mg/kg) post-transplantation.

### 2.2 Cell culture

The murine HCC cell line Hepa1-6 luc was obtained from Tianjin Medical University. The cells were authenticated by STR profiling and confirmed to be free of *mycoplasma* contamination. They were maintained in high-glucose Dulbecco’s Modified Eagle Medium supplemented with 10% fetal bovine serum.

### 2.3 Heterotopic heart transplantation

A mouse model of heterotopic heart transplantation was established by implanting donor hearts from Balb/c mice into the abdominal cavity of C57BL/6 recipients, as previously described (Kong et al., 2024b). Briefly, hearts were harvested from Balb/c mice under isoflurane anesthesia. The aorta and pulmonary artery of the donor heart were anastomosed to the abdominal aorta and inferior vena cava of the recipient mouse, respectively. Graft survival was assessed by daily palpation of abdominal cardiac pulsation.

### 2.4 Histology

Samples were fixed in 10% formalin for 48 h, embedded in paraffin, and sectioned at 5 μm intervals for hematoxylin-eosin (H&E) staining to facilitate histopathological confirmation of tumor formation and evaluation of the severity of allograft rejection. As previously described, allograft rejection was evaluated based on the presence of lymphocyte infiltration, vasculitis, infarction, myocyte necrosis, intravascular thrombosis, and interstitial hemorrhage. Specifically, compared to normal tissue, the severity of each pathological feature was scored as follows: 0, no change; 1, minimal change; 2, mild change; 3, moderate change; and 4, marked change. For each cardiac allograft, three fields were randomly selected and scored (Kong et al., 2024b).

### 2.5 *In vivo* imaging

The ventral side of the mice was depilated to prevent interference during imaging. Then, the mice were anesthetized with isoflurane. 10 min before *in vivo* imaging, D-luciferin sodium (Mce, HY-12591) dissolved in PBS was intraperitoneally injected into mice at a dose of 150 mg/kg. To prevent interference from non-specific luminescence resulting from skin abrasion, the mice’s abdomen and tail were covered with black cardboard. Bioluminescence imaging was performed via *in vivo* imaging system 10 min after injection.

### 2.6 Measurement of serum alpha-fetoprotein and cytokines levels

Collected peripheral blood samples were allowed to stand at room temperature for 30 min, followed by centrifugation at 3000 rpm for 20 min to isolate the serum. Serum alpha-fetoprotein (AFP) levels were measured using the Mouse AFP ELISA Kit (Elabscience, E-EL-M2405), and the serum levels of IL-2, IL-4, IL-6, IL-10, IL-17a, IFN-γ, and TNF-α were measured using the ABplex Mouse 9-Plex Custom Panel (ABclonal, RK04383), according to the manufacturer’s instructions.

### 2.7 Statistics

All statistical analyses were performed using GraphPad Prism (version 9.5). Data are expressed as mean ± SD. Comparisons among multiple groups were conducted by one-way ANOVA. Graft survival was assessed using Kaplan–Meier curves, and comparison of survival differences among groups was performed with the log‐rank (Mantel-Cox) test. A *p*-value of less than 0.05 was considered statistically significant.

## 3 Results

### 3.1 Establishment of the mouse model of post-transplant HCC recurrence

**FIGURE 1:**
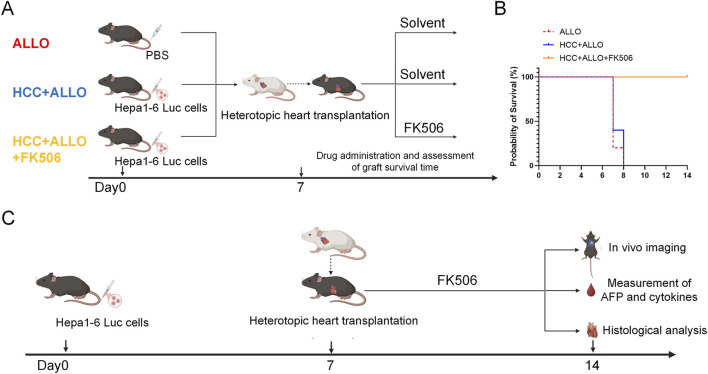
Schematic of the model establishment. **(A)** Schematic of the experimental design for cardiac allograft survival assessment. **(B)** Kaplan–Meier survival curves of cardiac allografts in the different experimental groups. **(C)** Schematic of the mouse model for post-transplant HCC recurrence. (n = 5).

To define the optimal time point for monitoring allograft rejection and tumor burden, we first assessed cardiac graft survival times in the ALLO, HCC + ALLO, and HCC + ALLO + FK506 groups (Figure 1A). As shown in Figure 1B, the graft survival time was 7–8 days in both the ALLO and HCC + ALLO groups, whereas it exceeded 14 days in the HCC + ALLO + FK506 group. To model the immunological conflict between graft rejection and cancer progression, it was essential to ensure concurrent graft survival and the presence of tumors. Therefore, the assessment of tumor burden in recipients and graft rejection should be performed on day 7 post-transplantation, and we established the mouse model of post-transplantation cancer recurrence as Figure 1C. C57BL/6 mice were randomly assigned to four groups: HCC group, ALLO group, HCC + ALLO group, and HCC + ALLO + FK506 group. On Day 14, corresponding to day 7 post-transplantation, tumor formation was detected via *in vivo* imaging. Subsequently, peripheral blood, cardiac allografts, and native organs from the recipients were collected. Body weight changes in each group of mice are shown in Supplementary Figure 1. Mice in the ALLO, HCC + ALLO, and HCC + ALLO + FK506 groups exhibited a decrease in body weight after heart transplantation, but the decrease was less than 20% in all cases. Furthermore, no significant abnormalities were observed in the appearance, food and water intake, or voluntary activity of mice in any group.

### 3.2 Evaluation of cardiac allograft rejection

**FIGURE 2:**
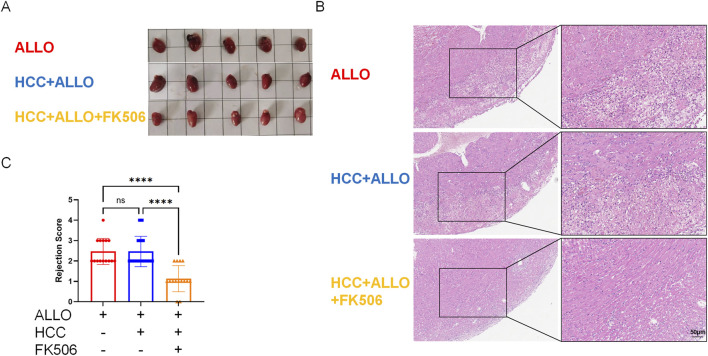
FK506 attenuates acute cardiac allograft rejection in mice. **(A)** Gross observation of allografts in each groups. **(B)** Histopathological changes of cardiac allografts are assessed by H&E staining. **(C)** Bar graph showing the rejection scores of cardiac allografts in each group. Statistical analysis was conducted by one-way ANOVA (n = 5). Data are shown as mean ± SD; ns, *P* > 0.05, *P < 0.05, **P < 0.01, ***P < 0.001, ****P < 0.0001.

As shown in Figure 2A, gross observation revealed that the cardiac allografts in both the ALLO group and the HCC + ALLO group appeared dull and dark red, with a slightly enlarged volume compared to normal hearts. In contrast, the cardiac allografts in the HCC + ALLO + FK506 group exhibited a glossy surface, and their color and size were nearly identical to those of normal hearts. H&E staining revealed severe acute rejection in the cardiac allografts of both the ALLO and HCC + ALLO groups, characterized by inflammatory cell infiltration, myocardial hemorrhage, and disruption of tissue architecture. Treatment with FK506 notably attenuated allograft damage and resulted in a significantly lower rejection score compared to the HCC + ALLO group (*P* < 0.0001) (Figures 2B,C). These results collectively demonstrate that our model faithfully recapitulates the clinically observed efficacy of FK506 in alleviating graft rejection.

### 3.3 Analysis of serum cytokine levels

**FIGURE 3:**
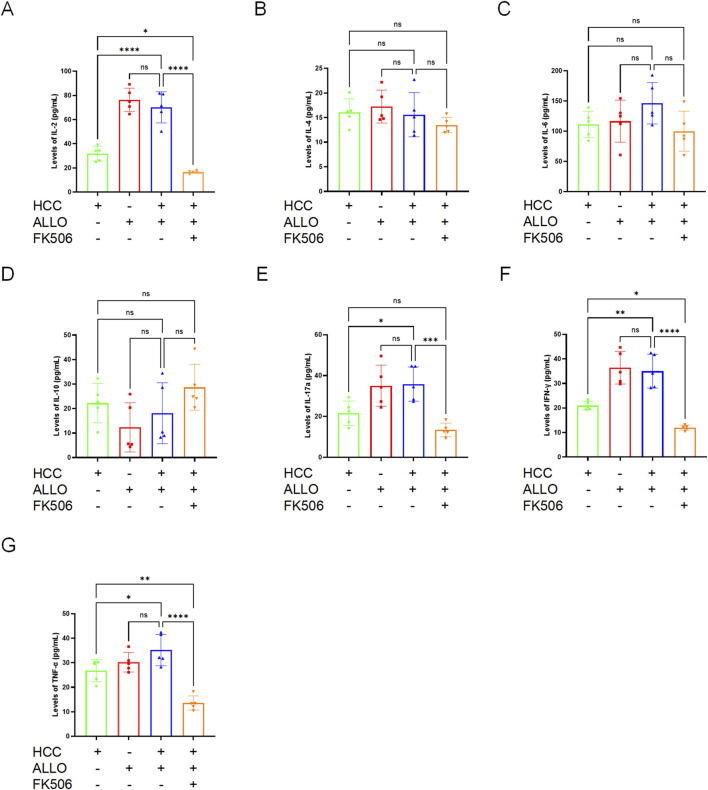
FK506 induces immunosuppression in mice following transplantation. **(A–G)** Concentrations of the indicated cytokines were measured in serum collected on day 7 after heart transplantation. Statistical analysis was conducted by one-way ANOVA (n = 5). Data are shown as mean ± SD; ns, *P* > 0.05, *P < 0.05, **P < 0.01, ***P < 0.001, ****P < 0.0001.

Cytokines serve as key indicators of immune system state, therefore, we measured serum cytokine levels in mice from each experimental group. As shown in Figure 3, mice in the HCC + ALLO group exhibited significantly elevated levels of pro-inflammatory cytokines (IL-2, *P* < 0.0001; IL-17a, *P* < 0.05; IFN-γ, *P* < 0.01; TNF-α, *P* < 0.05) relative to the HCC group, indicating a potent activation of the recipient’s immune system by the allograft. In contrast, FK506 treatment effectively abolished this increase. The levels of IL-2 (*P* < 0.05), IFN-γ (*P* < 0.05), and TNF-α (*P* < 0.01) were even lower than those in the HCC group. Additionally, no significant differences were observed in the levels of IL-4, IL-6 and IL-10 across the experimental groups. Collectively, these results indicate that our model successfully recapitulates the FK506-mediated immunosuppressive state observed in clinical transplantation.

### 3.4 Assessment of tumor burden post-transplantation

**FIGURE 4:**
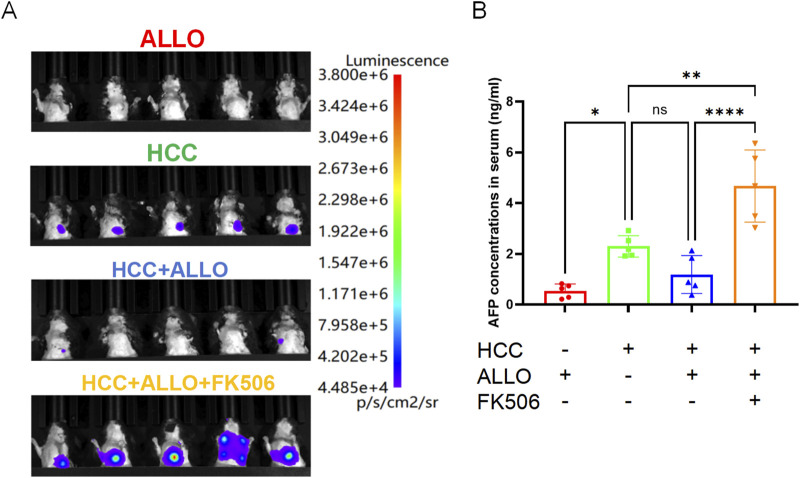
FK506 significantly elevates the risk of HCC recurrence after transplantation. **(A)** Assessment of HCC tumor burden in recipient mice by *in vivo* imaging. **(B)** Serum AFP levels reflect the HCC tumor burden in recipient mice. Statistical analysis was conducted by one-way ANOVA (n = 5). Data are shown as mean ± SD; ns, *P* > 0.05, *P < 0.05, **P < 0.01, ***P < 0.001, ****P < 0.0001.

**FIGURE 5:**
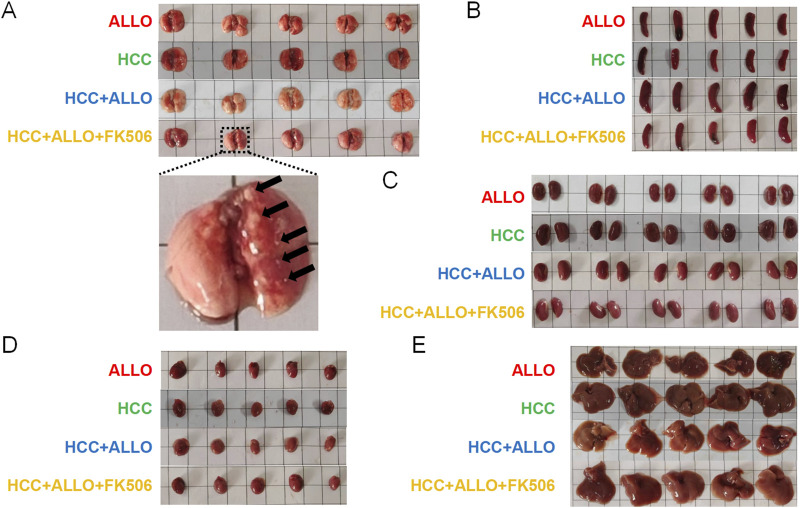
Gross observation of the major organs. **(A)** Gross observation of the lungs. **(B)** Gross observation of the spleens. **(C)** Gross observation of the kidneys. **(D)** Gross observation of the hearts. **(E)** Gross observation of the livers. (n = 5).

**FIGURE 6:**
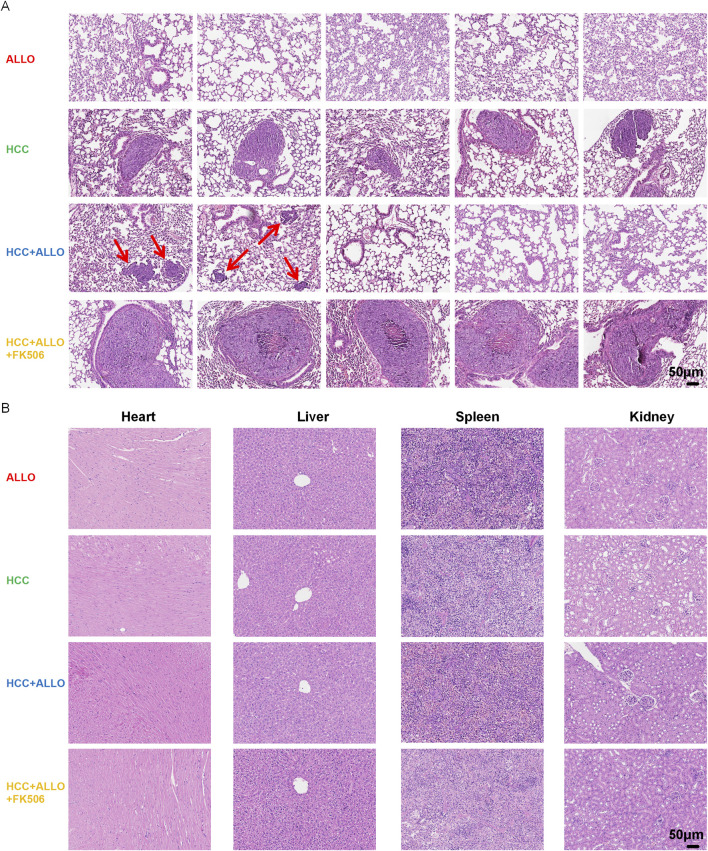
Histological examination of HCC metastasis in major organs. **(A)** Detection of HCC metastasis in the lungs of each group by H&E staining. **(B)** H&E staining revealed no HCC tumor foci in the heart, liver, spleen, or kidneys across all groups. (n = 5).

As shown in Figure 4A, *in vivo* imaging revealed stable bioluminescent signals in all mice of the HCC group by Day 14, confirming established tumor growth. In contrast, only 40% (2/5) of the animals in the ALLO + HCC group exhibited faint detectable signals. This phenomenon, combined with the observed post-transplant increase in multiple pro-inflammatory cytokines, suggests that the allograft activated the recipient’s immune system, leading to enhanced anti-tumor cytotoxicity. Conversely, all mice in the HCC + ALLO + FK506 group displayed intense luminescence, corroborating the tumor-promoting role of this first-line immunosuppressant. To further evaluate the HCC burden, serum AFP levels were measured. As depicted in Figure 4B, AFP levels in the HCC group were significantly higher than in the tumor-free ALLO group (*P* < 0.05). Notably, the HCC + ALLO + FK506 group exhibited a marked increase in AFP levels relative to both the HCC (*P* < 0.01) and HCC + ALLO group (4*P* < 0.0001). Gross inspection revealed that tumor nodules were solely confined to the lungs (Figure 5A), with no macroscopic lesions observed in the spleens (Figure 5B), kidneys (Figure 5C), hearts (Figure 5D) or livers (Figure 5E). Consistent with the *in vivo* imaging findings, H&E staining confirmed that the pulmonary metastatic incidence was 100% (5/5) in both the HCC and HCC + ALLO + FK506 groups, but only 40% (2/5) in the HCC + ALLO group (Figure 6A). No metastases were identified in other examined organs, including the heart, liver, spleen, and kidneys, across all groups (Figure 6B). Taken together, although FK506 effectively attenuates allograft rejection, the immunosuppressive state it induced was associated with an elevated risk of malignancy in the recipient.

## 4 Discussion

Organ transplantation is complicated by a dual risk of malignancy and graft rejection, presenting a significant clinical challenge, particularly for patients undergoing liver transplantation for HCC. Although first-line immunosuppressants such as the calcineurin inhibitor FK506 are effective in preventing rejection, prolonged immunosuppression significantly elevates the risk of HCC recurrence (van der Meeren et al., 2025). Therefore, a critical unmet need in HCC liver transplantation is how to balance immunosuppression to minimize rejection while concurrently reducing the risk of HCC recurrence. Researchers have developed various cancer-transplant animal models to investigate the mechanisms underlying and prevention strategies for HCC recurrence following liver transplantation. However, these existing models fail to meet one or several of the following key factors required for mimicking clinical post-transplant HCC recurrence: an immunosuppressed state, an authentic tumor microenvironment, a vascularized allograft and stability (Table 1).

**TABLE 1:** Existing cancer-transplant animal models.

Tumor Induction method	Transplant model	Immunosuppression	Transplant timing	Referrence
DEN	Fischer→Fischer rat, liver	+	After tumor	Matsuzaki et al. (1992)
Intrahepatic implantation	Lewis→ACI rat, liver	+	After tumor	Yan et al. (2013)
DEN	BN→BN rat, liver	-	After tumor	Schotman et al. (1998)
DEN	SD→SD rat, liver	-	After tumor	Li et al. (2004)
DEN	Lewis→Lewis rat, liver	-	After tumor	Ceriello et al. (1994)
DEN	Buffalo→Buffalo rat, liver	-	After tumor	Yano et al. (1998)
Intrahepatic implantation	Buffalo→Buffalo rat, liver	-	After tumor	Ushitora et al. (2009)
Subcutaneously injection	BALB/c→C57BL/6 mouse, skin	-	After tumor	Yang et al. (2023)
Subcutaneously injection	BALB/c→C57BL/6 mouse, heart	+	After tumor	Koehl et al. (2004)
Portal vein injection	Buffalo→Buffalo rat, liver	-	Before tumor	Qi et al. (2016), Liu et al. (2021), Li et al. (2016), Oldani et al. (2019)
Portal vein injection	Fischer→Fischer rat, liver	-	Before tumor	Oldani et al. (2014)
Portal vein injection	Wistar→Wistar rat, liver	-	Before tumor	Wang et al. (2017)

Abbreviations: DEN, diethylnitrosamine.

Although orthotopic liver transplantation in HCC animal models can replicate the natural history of post-transplant recurrence, the process is protracted and exhibits poor stability (Schotman et al., 1998; Li et al., 2004; Ceriello et al., 1994; Yano et al., 1998), even under immunosuppression (Matsuzaki et al., 1992; Yan et al., 2013). This is primarily because the intravasation of orthotopic HCC cells into the circulation is a complex process, resulting in circulating tumor cells (CTCs) at a low and variable frequency. Therefore, to establish a more rapid and consistent model of HCC recurrence, we directly injected a defined number of HCC cells to generate recurrent foci.

In existing models, tumors are established by portal vein injection to form liver tumors or by subcutaneous implantation. However, the most common site of HCC recurrence after clinical liver transplantation is the lungs (Alshahrani et al., 2018; Na et al., 2016; de'Angelis et al., 2015). Mechanistically, CTCs that intravasate from the primary HCC site into the bloodstream are the primary cause of pulmonary metastasis after liver transplantation (Chen et al., 2024). After entering the bloodstream, circulating HCC cells evade immune surveillance and, via the inferior vena cava and right heart, first reach the lungs where they lodge and colonize. It is noteworthy that due to differences in immune cell composition, vascular architecture, and oxygenation levels, the tumor microenvironment in the liver and subcutaneous sites is markedly distinct from that in the lung, which inevitably leads to divergent responses to therapeutic interventions. Tail vein injection recapitulates the clinically relevant process of HCC lung metastasis initiated by CTCs. Consequently, we made the strategic decision to inject HCC cells via the tail vein before transplantation to establish a model of pulmonary recurrence.

As for allograft, while the murine allogeneic skin transplantation model is operationally simple, its rejection mechanisms differ from those of vascularized solid organ transplantation. Vascular-related rejection plays a central role throughout the continuum of solid organ transplant rejection. The donor endothelium, upon immediate contact with the recipient’s blood, serves as a primary target—initiating hyperacute rejection via preformed antibodies, driving acute rejection by alloreactive T and B cells, and progressively leading to chronic graft vasculopathy (Masutani, 2023). These vessel-centered processes are fundamental to clinical transplant pathology, yet are entirely absent in non-vascularized skin grafts. As for the mouse liver transplantation model, its stability is often compromised due to factors such as spontaneous tolerance, and its prohibitive technical difficulty further limits widespread adoption. The Balb/c-to-C57BL/6 mouse abdominal heterotopic heart transplantation is the most commonly used model for investigating rejection mechanisms and evaluating immunosuppressants, as it allows for simple assessment of graft survival by palpation and exhibits high reproducibility (Kong et al., 2023).

The complex immune state of the recipient comprises both the activation of the immune system by alloantigens and a more profound suppression mediated by immunosuppressants. Following transplantation, antigen-presenting cells capture alloantigens from the graft and present them to naïve CD4^+^ T cells, driving their differentiation into subsets such as Th1, Th2, and Th17. Th1 cells secrete pro-inflammatory cytokines, including IL-2, IFN-γ, and TNF-α, which mediate graft rejection by activating cytotoxic T lymphocytes and macrophages; Th17 cells contribute to graft damage primarily by releasing IL-17, which recruits neutrophils to the site (Ren et al., 2024). Current cornerstone immunosuppressants, such as FK506, alleviate rejection primarily by inhibiting T-cell activation and proliferation, as well as cytokine production. However, this simultaneously impairs immune surveillance against tumor cells, thereby significantly increasing the risk of HCC recurrence in recipients. To model the complex immune state of clinical transplant recipients, we administered FK506 daily to induce an immunosuppressed state in the recipients to promote the progression of HCC.

Considering these key factors, we finally established a model that closely recapitulates clinical post-transplant HCC pulmonary recurrence by combining tail vein injection of Hepa1-6 luc cells with FK506 treatment to attenuate allograft rejection in C57BL/6 mice receiving cardiac allografts from Balb/c. This model recapitulates the formation of pulmonary metastases from pre-existing circulating HCC cells under the immunosuppressed state following transplantation. To fully capture the immunological conflict between the allograft and HCC, we first determined cardiac graft survival across experimental groups, which identified post-operative day 7 as the optimal time point for simultaneous assessment of both rejection and HCC burden. Our integrated analyses—histology for rejection, serum cytokines for systemic immune state, and *in vivo* imaging combined with serum AFP for tumor burden—collectively demonstrated that allogeneic transplantation significantly reduced tumor burden. This phenomenon is likely attributable to allograft-induced systemic immune activation, as evidenced by a marked post-transplant increase in serum levels of IL-2, IFN-γ, and TNF-α, which are pivotal cytokines in adaptive anti-tumor immunity (Kureshi and Dougan, 2025). In contrast, FK506 created a profoundly immunosuppressed state in recipients, as evidenced by a marked decrease in serum levels of IL-2, IFN-γ, and TNF-α, which alleviated allograft rejection but concurrently promoted pulmonary HCC recurrence.

Notably, immunosuppressants with concomitant antitumor effects represent a superior strategy for preventing HCC recurrence following transplantation. For instance, inhibitors of the mammalian target of rapamycin (mTOR), exert dual antitumor and immunosuppressive effects by blocking the mTOR pathway (Li et al., 2014). Immunosuppressive regimen combining an mTOR inhibitor with a reduced-dose of FK506 may contribute to a lower recurrence rate of HCC in recipients (Lee et al., 2021). However, concerns regarding adverse effects such as hepatic artery thrombosis and delayed wound healing have limited its early post-operative application (Asrani et al., 2014). This model is highly suitable for evaluating novel immunosuppressants that possess dual anti-tumor and immunomodulatory functions. Furthermore, exploring targets within T cells that suppress allograft rejection while preserving anti-tumor immunity holds significant clinical value for organ transplantation (Tan et al., 2025), this model is suitable for validating such targets.

The limitation is that the technical challenges and low stability associated with mouse orthotopic liver transplantation led us to employ heart allografts to investigate the relationship between the post-transplant immunosuppression and HCC recurrence. However, hepatic resident immune cells (including dendritic cells, T cells, Kupffer cells, and potentially NK cells) communicate with parenchymal liver sinusoidal endothelial cells and hepatocytes, in conjunction with specific anti-inflammatory cytokines and signaling molecules, to establish a tolerogenic microenvironment within the liver (Dai et al., 2020). Therefore, the mouse cardiac transplantation model cannot fully recapitulate the rejection mechanisms observed in liver transplantation and is characterized by more vigorous rejection. This model is thus unsuitable for studies investigating liver-specific immunotherapies. Furthermore, the relatively modest sample size (n = 5) employed in this study represents an additional limitation.

In summary, we have established a novel mouse model of post-transplant HCC pulmonary recurrence mediated by FK506 that recapitulates key clinical features, including the immunosuppressed state and the lung as the most common site of recurrence. This model serves as a reliable tool for developing preventive strategies against post-transplantation HCC recurrence.

## Data Availability

The raw data supporting the conclusions of this article will be made available by the authors, without undue reservation.

## Supplementary material

SUPPLEMENTARY FIGURE 1 Line graph of body weight changes in mice.
