# Supplementary figures and images for: Establishing a novel mouse model of tacrolimus-induced post-transplant hepatocellular carcinoma pulmonary recurrence for transplant oncology
Source: Front Cell Dev Biol. 2026 Apr 1;14:1796566. doi: 10.3389/fcell.2026.1796566 (PMC13079379; doi:10.3389/fcell.2026.1796566)

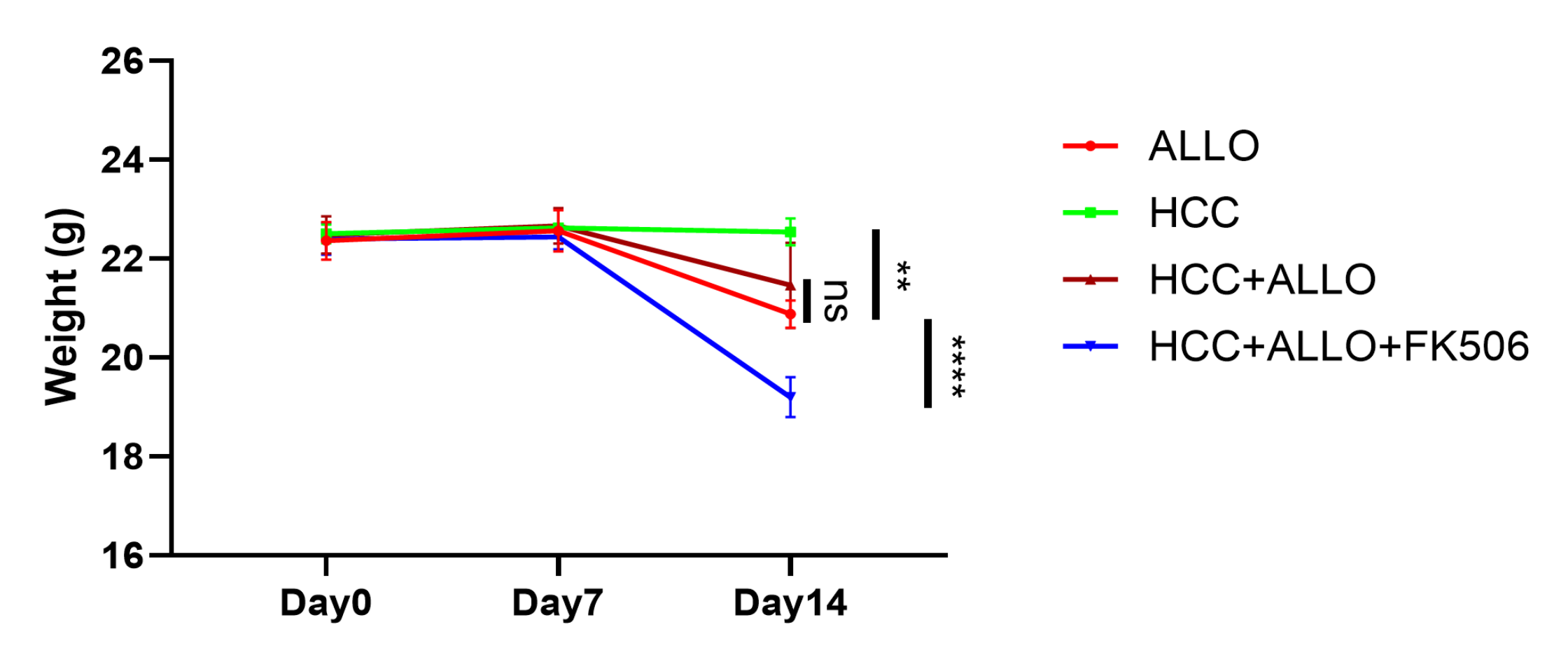

Supplement: Supplementary file 1 [file Image1.tif]
